# The Na/K-ATPase α1/Src Signaling Axis Regulates Mitochondrial Metabolic Function and Redox Signaling in Human iPSC-Derived Cardiomyocytes

**DOI:** 10.3390/biomedicines11123207

**Published:** 2023-12-02

**Authors:** Liquan Cai, Marco T. Pessoa, Yingnyu Gao, Sidney Strause, Moumita Banerjee, Jiang Tian, Zijian Xie, Sandrine V. Pierre

**Affiliations:** 1 Marshall Institute for Interdisciplinary Research, Marshall University, Huntington, WV 25703, USA; cail@marshall.edu (L.C.); correapessoa@marshall.edu (M.T.P.); gaoy@marshall.edu (Y.G.); strause@marshall.edu (S.S.); moumita.banerjee@uky.edu (M.B.); tianj@marshall.edu (J.T.); xiez@marshall.edu (Z.X.); 2 Markey Cancer Center, University of Kentucky, Lexington, KY 40536, USA; 3 Department of Surgery, University of Kentucky, Lexington, KY 40536, USA; 4 Joan C. Edwards School of Medicine, Marshall University, Huntington, WV 25701, USA

**Keywords:** ATP1A1, Src, human induced pluripotent stem cell, mitochondrial metabolic function, cardiac metabolism, oxidative stress

## Abstract

Na/K-ATPase (NKA)-mediated regulation of Src kinase, which involves defined amino acid sequences of the NKA α1 polypeptide, has emerged as a novel regulatory mechanism of mitochondrial function in metazoans. Mitochondrial metabolism ensures adequate myocardial performance and adaptation to physiological demand. It is also a critical cellular determinant of cardiac repair and remodeling. To assess the impact of the proposed NKA/Src regulatory axis on cardiac mitochondrial metabolic function, we used a gene targeting approach in human cardiac myocytes. Human induced pluripotent stem cells (hiPSC) expressing an Src-signaling null mutant (A420P) form of the NKA α1 polypeptide were generated using CRISPR/Cas9-mediated genome editing. Total cellular Na/K-ATPase activity remained unchanged in A420P compared to the wild type (WT) hiPSC, but baseline phosphorylation levels of Src and ERK1/2 were drastically reduced. Both WT and A420P mutant hiPSC readily differentiated into cardiac myocytes (iCM), as evidenced by marker gene expression, spontaneous cell contraction, and subcellular striations. Total NKA α1-3 protein expression was comparable in WT and A420P iCM. However, live cell metabolism assessed functionally by Seahorse extracellular flux analysis revealed significant reductions in both basal and maximal rates of mitochondrial respiration, spare respiratory capacity, ATP production, and coupling efficiency. A significant reduction in ROS production was detected by fluorescence imaging in live cells, and confirmed by decreased cellular protein carbonylation levels in A420P iCM. Taken together, these data provide genetic evidence for a role of NKA α1/Src in the tonic stimulation of basal mitochondrial metabolism and ROS production in human cardiac myocytes. This signaling axis in cardiac myocytes may provide a new approach to counteract mitochondrial dysfunction in cardiometabolic diseases.

## 1. Introduction

Through its enzymatic cycle, the sarcolemmal Na/K-ATPase (NKA) undergoes conformational transitions and utilizes ATP hydrolysis to power the extrusion of 3 Na^+^ against 2 K^+^. This fundamental function of NKA has long been studied and targeted pharmacologically for its critical role in cellular ion homeostasis during cardiac contraction-relaxation [1,2,3]. Most notably, it is well established that reduction of Na^+^ extrusion through partial inhibition of cardiac NKA augments cardiac contractility by modulating Na^+^/Ca^2+^-exchange and subsequently increasing intracellular Ca^2+^ [4,5].

Experimental evidence of additional properties of the sarcolemmal NKA, collectively known as signaling functions, were first reported two decades ago by Peng et al. and Huang et al. in rat neonatal cardiac myocytes [6,7]. Analogous observations have since been made in numerous cell types, organs, and organisms, revealing that prevalent cell-specific scaffolds, protein kinases, and second messengers of the NKA axis interface with major cell regulatory signaling pathways in health and disease [1,8,9,10,11,12,13,14,15,16,17,18,19,20,21,22,23,24,25,26,27,28,29,30,31,32,33]. Among those, NKA-dependent modulations of Src/EGFR, reactive oxygen species (ROS), and PI3K/Akt cascades have been the main focus of investigation in the cardiac myocyte (CM) [1].

Both ion-transport and signaling functions require functional assembly of NKA α and β subunit oligomers at the sarcolemma. Cardiac NKAs are combinations of α1-3 and β1-2 isoforms, with species-specificity and highly regulated patterns of myocardial expression observed during ontology and development [2,3,34,35,36,37,38,39]. NKA isoforms α1-3 are expressed in human hearts [36,40], while α1 and α2 are the predominant forms found in the adult rodent heart [3,41]. Derangements of cardiac NKA isoforms have long been described in major cardiovascular diseases, including cardiomyopathies and heart failure [40,42,43,44,45,46], yet in most cases the underlying mechanisms and functional consequences of those changes remain incompletely understood. Clinically, we are just beginning to understand the role of NKA isoform gene polymorphisms in health and disease [47,48,49]. Experimentally, genetic approaches in mice have provided evidence for α-isoform-specific roles in cardiovascular physiology [50,51,52,53,54,55]. We have recently observed that selectively increasing the interaction between endogenous cardiotonic steroids (CTS) and NKA α1 isoform (via targeted mutation of *Atp1a1*) did not modify cardiac Na/K-ATPase activity, but significantly upregulated myocardial ROS and pathways related to metabolism/energy production (e.g., NADH dehydrogenase complex and respiratory chain, TCA cycle, respiratory electron transport, ATP synthesis, and mitochondrial fatty acid β-oxidation) [55]. Importantly, cardiac remodeling with structural and functional features of physiological hypertrophy developed spontaneously overtime in this model, suggestive of a profound biological impact of endogenous CTS/NKA signaling on the myocardium that has remained unexplored.

Based on recent reports from our group and others [47,56], we hypothesized that an isoform-specific regulatory mechanism downstream of the NKAα1/Src receptor sets mitochondrial metabolic output and oxidative signaling in cardiac muscle cells. This was explicitly tested using a CRISPR/Cas9-mediated genetic approach. Specifically, mitochondrial metabolic function and redox status were evaluated in induced CM derived from human induced pluripotent stem cells (hiPSC) expressing a wild type NKA α1 or an A420P mutant form of NKA α1 with altered NKA/Src-regulatory function.

## 2. Materials and Methods

### 2.1. Human Induced Pluripotent Stem Cells (hiPSC): WT and A420P Mutant Lines

Human iPSC were purchased from iXCells Biotechnologies (San Diego, USA, #30HU-002) and cultured, as we have previously described [57], in maintenance media from the TeSR™-E8™ Kit (STEMCELL Technologies Inc., Vancouver, BC, Canada, #05990) on Geltrex (Thermo Fisher Scientific Inc., Waltham, MA, USA, #A1413201) pre-coated plates. The A420P-NKA α1 mutant hiPSC line was chosen as a well-characterized NKA/Src loss-of-function mutation [58,59]. As we have reported recently, hiPSC-A420P were generated using CRISPR-Cas9 genome editing [57]. A TseI restriction site was introduced for clone selection. A genotyping PCR amplified the DNA sequence flanking the target region from the genomic DNA of WT vs. three A420P mutant iPSCs clones, which were further validated by DNA sequencing.

### 2.2. Differentiation of hiPSC into Cardiomyocytes (iCM)

We used the STEMdiff™ Ventricular Cardiomyocyte Differentiation Kit (STEMCELL, Vancouver, BC, Canada, #05010), which consists of Differentiation Media A, B, C, and Maintenance Medium. Induction of differentiation was conducted according to the manufacturer’s instructions. Human iPSC were dissociated with TrypLE (Thermo Fisher Scientific Inc., Waltham, MA, USA, #12605010) and grown on Matrigel (Corning, New York, NY, USA, #354234)-coated plates at a density of 2 × 10^5^ cells/cm^2^. At confluency, the culture medium was switched to Medium A plus Matrigel (1:100 ratio) for two days, followed by two days of Medium B, and two rounds of Medium C (two days each round). Lastly, the induced cardiomyocytes (iCM) were cultured in Maintenance Medium until further analysis. Beating cardiomyocytes were exclusively selected for further studies.

### 2.3. Immunostaining and Fluorescence Microscopy

Cultured iCM were stained and visualized according to an experimental protocol slightly modified from the one we have recently reported in human induced skeletal muscle cells [57]. First, iCM initially grown in clustered sheets were digested into single cells with Accutase, (Innovative Cell Technologies, Inc., San Diego, CA, USA, #AT104), filtered with 70 µm cell strainers (Genesee Scientific Corporation, #25-376), and seeded in a 6-well plate where sterilized coverslips were placed and pre-coated with Matrigel, as above. Following cell fixation in prechilled methanol for 15 min and blocking with 3% horse serum, iCM were incubated with anti-sarcomeric α-actinin (Abcam, Waltham, MA, USA, #ab9465—RRID:AB 307264, 1:200 dilution) at 4 °C overnight. Next, the cells were incubated with a chicken anti-mouse IgG Alexa Fluor^TM^ 488 secondary antibody (Thermo Fisher Scientific Inc., Waltham, MA, USA, #A21200, 1:200 dilution) for one hour at room temperature and counterstained with DAPI. We used a fluorescence microscope and took pictures from at least three random fields per slide. At least five slides were mounted from independent experiments. Positive staining for α-actinin was in the range of 60–70% for both WT and A420P iCM, as determined by the ratio of positive stained cells/total cell number estimated by DAPI-only stained cells. An investigator blinded to the cell genotype performed all the analyses.

### 2.4. Video imaging

Beating iCM were imaged with a 10× objective lens under bright-field illumination at room temperature using a Leica DFC310FX camera (Leica Camera Inc., Teaneck, NJ, USA) (gain: 2, 2 × 2 binning, exposure time: 10 ms, 8-bit greyscale mode with 10 frames per second image acquisition). Videos were ultimately exported with a frame rate of 20 frames per second.

### 2.5. Electron Microscopy Imaging

The striation of iCM was visualized under electron microscopy (EM), as we have described previously [57]. Briefly, iCM were fixed with glutaraldehyde and paraformaldehyde and embedded in epoxy. Sections were cut at approximately 90 nm thickness, stained with lead citrate, and imaged on an FEI Techai 12 using a Gatan camera and software.

### 2.6. RNA Purification, Reverse Transcription, and Real-Time qPCR

Total RNA from hiPSC and iCM was isolated using TRIzol reagent (Thermo Fisher Scientific Inc., Waltham, MA, USA, #15596018). Total RNA samples (1 μg) with a concentration > 20 ng/μL were examined for quality control, including OD260/280 > 2.0 (Table S1) and transcribed into cDNA using the SuperScript^®^ III First-Strand Synthesis system (Thermo Fisher Scientific Inc., Waltham, MA, USA, #11752-050). Next, the cDNA was amplified in a 384-microwell plate using a LightCycler^®^ 480 Real-Time PCR System and SYBR Green master mix (Roche, Indianapolis, IN, USA, #4887352001). The relative expression of a specific gene was calculated according to the 2 (−ΔΔCt) method [60,61]. Hypoxanthine-guanine phosphoribosyltransferase 1 gene (*HPRT1*) was used as the reference gene. The primer sequences are listed in Table S2.

### 2.7. Ouabain-sensitive Na/K-ATPase Activity

As described by Lai et al. [58], cells were collected and homogenized in ice-cold buffer A (250 mM sucrose, 30 mM histidine, and 1 mM EDTA, pH 7.4) and briefly sonicated (for 10 s, 3 times, with a 10 s interval at 15% amplitude). After centrifugation (800 × *g* for 10 min), the supernatant was further centrifuged (125,000 × *g* for 45 min) to obtain a membrane preparation. The membrane pellet was resuspended in buffer A, and the protein content was determined. Resuspended crude membranes were treated with alamethicin (MilliporeSigma, Burlington, VT, USA, #A-4665) (0.1 mg/mg of protein) for 10 min at room temperature and then added to pre-warmed buffer containing 20 mM Tris (pH 7.2), 1 mM MgCl_2_, 100 mM NaCl, 1 mM EGTA, 5 mM NaN_3_, and 20 mM KCl. A set of tubes contained the Na/K-ATPase specific inhibitor ouabain (MilliporeSigma, Burlington, VT, USA, #O3125) at 1 mM. After 10 min of preincubation at 37 °C, ATP (MilliporeSigma, Burlington, VT, USA, #A9062)/Mg^2+^ was added to a final concentration of 2 mM to start the reaction. The reaction continued for 45 min and was stopped by adding 8% ice-cold trichloroacetic acid. The inorganic phosphate generated during the ATP hydrolysis was measured using the BIOMOL Green Reagent (Enzo Life Science, New York, NY, USA, #BML-AK111). Ouabain-sensitive Na/K-ATPase activity was calculated as the difference between the values obtained in the presence or absence of 1 mM ouabain.

### 2.8. Cell Lysis and Western Blotting

Cell lysis, protein concentration measurement, and Western blot analyses were performed as described [35]. Human iPSC or iCM were digested into single cells with Accutase, (Innovative Cell Technologies, Inc., San Diego, CA, USA, #AT104), filtered with 70 µm cell strainers (Genesee Scientific Corporation, El Cajon, CA, USA, #25-376), centrifuged, and cell pellets were resuspended in modified ice-cold radioimmunoprecipitation assay buffer (RIPA) containing: 50 mM Tris-HCl (pH 7.4), 1% Nonidet P-40, 0.25% sodium deoxycholate, 150 mM NaCl, 1 mM EDTA, 1 mM phenylmethylsulfonyl fluoride, 1 mM Na_3_VO_4_, 1 mM NaF, and 1 μg/mL protease inhibitor cocktail (MilliporeSigma, Burlington, VT, USA, #P-8340). The cell lysates were centrifuged at 14,000 × *g* for 15 min at 4 °C, and the supernatants were separated by SDS-PAGE prior to transfer onto nitrocellulose membranes. After blocking with 5% non-fat dry milk, the membranes were probed with the following specific primary antibodies: anti-phospho ERK1/2 Thr202/Tyr204 (Cell Signaling Technology, Inc., Danvers, MA, USA, #9101—RRID:AB_331646, 1:1000 dilution); anti-ERK1 (Santa Cruz Biotechnology, Dallas, TX, USA, #sc-94, 1:1000 dilution); anti-phospho-Src Y419 (Thermo Fisher Scientific Inc., Waltham, MA, USA, #44-660G—RRID:AB_2533714, 1:1000 dilution); anti-c-Src (Santa Cruz Biotechnology, Dallas, TX, USA, #sc-8056—RRID:AB_627306, 1:1000 dilution); anti-Na/K-ATPase α1 (Developmental Studies Hybridoma Bank, Iowa City, IA, USA, #a6F—RRID:AB_528092, 1:100 dilution); HERED anti-Na/K-ATPase α2 (a gift from Dr. Presley, Texas Tech University, Lubbock, TX, USA, 1:200 dilution); TED anti-Na/K-ATPase α3 (a gift from Dr. Pressley, 1:500 dilution); Na/K-ATPase β1 (Santa Cruz Biotechnology, Dallas, TX, USA, Cat# sc-25709—RRID:AB_2060996, 1:500 dilution); and GAPDH (Abcam, Waltham, MA, USA, #ab181602—RRID:AB_2630358, 1:1000 dilution). Membranes were next incubated with species-specific HRP-conjugated secondary antibodies at room temperature for 1 h (anti-rabbit: R&D systems, Minneapolis, MN, USA, #HAF008—RRID:AB_357235, 1:1000 dilution; anti-mouse: Santa Cruz Biotechnology, Dallas, TX, USA, #sc-516102—RRID:AB_2687626, 1:1000 dilution). Membranes were exposed to ECL SuperSignal West Atto Ultimate Sensitivity Chemiluminescent Substract (Thermo Fisher Scientific Inc., #A38555) for Na/K-ATPase α1, α2, α3, and β1 isoforms or Pierce ECL Western Blotting Substrate (Thermo Fisher Scientific Inc., #32106) for GAPDH. Chemiluminescence was captured using a ChemiDoc Imaging System (Bio-Rad Laboratories, Inc., Hercules, CA USA). UltraCruz^TM^ autoradiography films (Santa Cruz Biotechnology, Dallas, TX, USA) were used to develop membranes for phospho-ERK1/2, ERK1, phosphor-Src and c-Src. Densitometry quantifications were performed using ImageJ 1.54f.

### 2.9. Seahorse Extracellular Flux Analysis 

Seahorse extracellular flux analysis was performed as previously described [56], with minor modifications. Briefly, iCM were plated at a 25,000 cells/well density on Seahorse XFp cell culture mini plates (Agilent Technologies, Inc., Santa Clara, CA, USA, #103025-100) pre-coated with Matrigel. Three days later, the iCMs were subjected to the Cell Mito Stress Test Kit (Agilent Technologies, Inc., Santa Clara, CA, USA, #103010-100), using an Agilent Seahorse XFp analyzer. For each assay, the order of injection, volumes, and concentrations of the respective compounds are listed in Table S3. Respiratory parameters were calculated according to the manufacturer’s instructions and included basal respiration, maximal respiration, spare respiratory capacity, ATP production, and coupling efficiency.

### 2.10. Protein Carbonylation 

Protein carbonylation was measured in whole cell lysates using the BioCell Protein Carbonyl Assay kit (BioCell Corporation, Auckland, New Zealand, #BPCK01). Derivatization of proteins from whole cell lysates was carried out according to the manufacturer’s instructions and protein carbonylation was estimated by comparing OD (450 nm) values to those of oxidized protein standards and an internal carbonyl control.

### 2.11. Detection of Reactive Oxygen Species (ROS)

Induced CM were plated in 96-well plates at 50,000 cells/well and cultured for one week. To quantify ROS, a dichlorodihydrofluorescein diacetate-based cellular assay (Thermo Fisher Scientific Inc., #C6827) was used as instructed. Briefly, iCM were incubated with 1 μM CM-H_2_DCFDA (Thermo Fisher Scientific Inc., #C6827) for 30 min at 37 °C, washed with HBSS + Ca^2+^/Mg^2+^ (Thermo Fisher Scientific Inc., #14025-092), and incubated for 10 min with 50 μM Hoechst 33342 (Thermo Fisher Scientific Inc., #62249) for nuclear staining. Induced CM were then washed and imaged immediately with a fluorescence microscope. Images were taken from at least three different wells per group in three independent experiments. Pictures were taken at 0, 30, and 60 s after exposing cells to the fluorescent light. Quantification was done on the green channel after splitting the RGB original file using ImageJ 1.54f. The Rolling Ball Radius for background subtraction was set to 50.0 pixels. The mean gray value was calculated for the entire picture and normalized by the number of nuclei. Relative fluorescence was calculated in reference to iCM-WT at 0 s. The slope of the curve (ΔF/time) was determined by linear regression from each group.

### 2.12. Statistical Analysis

All data are expressed as mean ± standard error of the mean (SEM). Unpaired Student’s *t*-test was used to compare the mean of two groups. A probability value of *p* < 0.05 was set as the cut-off for statistical significance.

## 3. Results

### 3.1. Characterization of WT and A420P hiPSC-Derived Cardiomyocytes (iCM)

**Figure 1:**
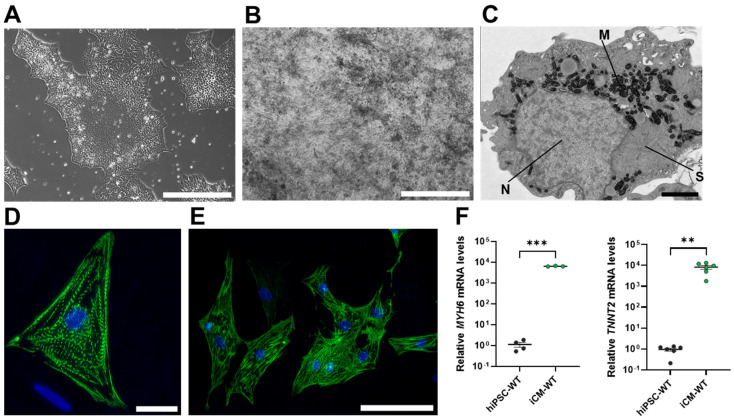
Generation of hiPSC-derived cardiomyocytes. (**A**) hiPSC colonies (scale bar: 200 µm). (**B**) Differentiated cardiomyocytes (iCM) (scale bar: 200 µm) contracted spontaneously (Video S1). (**C**) The differentiation protocol yielded cells highly enriched with mitochondria and typical muscle striation readily identifiable by electron microscopy (M: mitochondria, N: nucleus, S: muscle striation (scale bar: 2 µm). (**D**) Immunofluorescence staining showing typical cellular organization of cardiac α-actinin (scale bar: 25 µm). (**E**) Field view of positively stained iCM (scale bar: 100 µm). (**F**) Increased mRNA expression of marker genes myosin heavy chain 6 (*MYH6*) and cardiac troponin T2 (*TNNT2*) detected by RT-qPCR (*n* = 3–6/group. ** *p* < 0.01 and *** *p* < 0.001. Unpaired Student’s *t*-test). Pictures are representative of at least three independent experiments.

As shown in Figure 1, hiPSC-WT exposed to the protocol detailed in Section 2 differentiated into iCM (Figure 1A,B), characterized by spontaneous beating (Video S1), typical muscle striation evidenced by electron microscopy (Figure 1C), and expression of cardiac α-actinin (Figure 1D,E). In addition, iCM-WT expressed high mRNA levels of cardiac cell markers cardiac α-myosin heavy chain (*MYH6*) and cardiac troponin T2 (*TNNT2*) (Figure 1F) compared to the parent hiPSC-WT. Hence, iCM suitable to conduct the proposed studies of mitochondrial function and oxidative signaling were derived from hiPSC-WT cells using the protocol detailed in Section 2.

**Figure 2:**
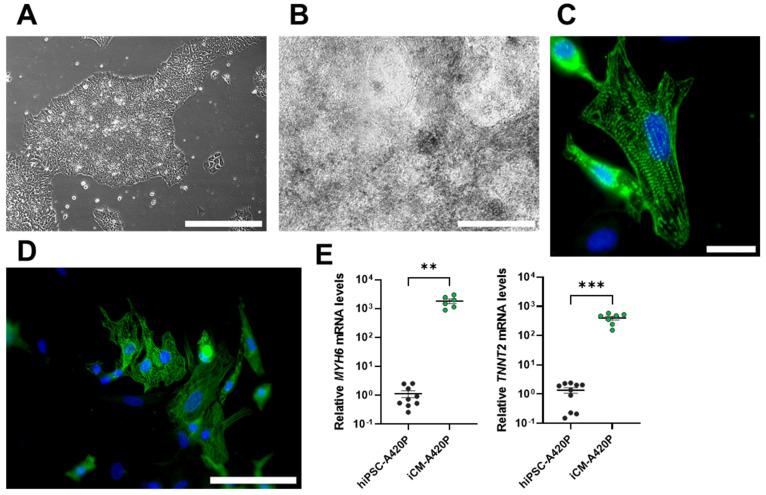
Induced A420P mutant cardiomyocytes. (**A**) hiPSC-A420P colonies (scale bar: 200 µm) were differentiated into iCM ((**B**) scale bar: 200 µm). (**C**) iCM-A420P have features indistinguishable from iCM-WT, including spontaneous contraction (Video S2), muscle striation revealed by positive immunostaining with an anti-sarcomeric α-actinin antibody (green) and nuclear counterstaining with DAPI (blue) (scale bar: 25 µm). (**D**) Field view of positively stained iCM-A420P (scale bar: 100 µm). (**E**) Upregulation of mRNA expression of cardiac markers α-myosin heavy chain (*MYH6*) and troponin T (*TNNT2*). *n* = 6–10/group, ** *p* < 0.01 and *** *p* < 0.001. Unpaired Student’s *t*-test. Pictures are representative of at least three independent experiments.

Next, to assess the role of NKA/Src in human cardiac cell mitochondrial metabolic function, we used hiPSC-A420P as a starting material and subjected them to the same iCM differentiation protocol. We have previously reported the generation of the mutant hiPSC-A420P line using CRISPR-Cas9 genome editing [57]. The A420P mutation targets the NaKtide sequence of the NKA α1 polypeptide, which, in turn, interferes with the NKA/Src receptor function of NKA α1-containing complexes in stable mutant epithelial cell lines [58]. Importantly, unlike another NKA-signaling mutant (CBM) which carries a defective N-terminal binding domain of the NKA α1 polypeptide, hiPSC-A420P retain the ability to differentiate into skeletal muscle [57], making them a potential candidate for differentiation into iCM. Consistent with previous observations, hiPSC-A420P formed colonies that were indistinguishable from the WT line (Figure 2A). At the end of the differentiation protocol, they presented overall cellular features of iCM (Figure 2B), spontaneous beating (Video S2), expression of sarcomeric α-actinin and muscle striation (Figure 2C-D), and increased mRNA levels of *MYH6* and *TNNT2* (Figure 2E). Accordingly, it was concluded that hiPSC-A420P’s ability to differentiate into iCM was indistinguishable from hiPSC-WT, an important pre-requisite for further evaluation.

### 3.2. Characterization of NKA Expression, Na^+^/K^+^-ATPase Activity, and Basal NKA Signaling Axis in A420P hiPSC and iCM

**Figure 3:**
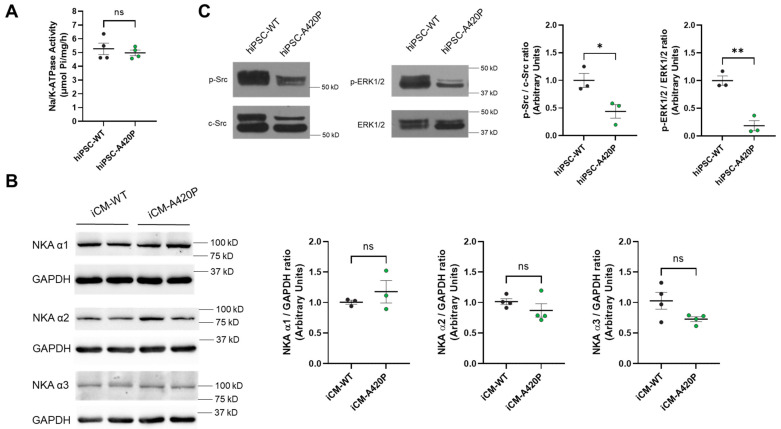
Characterization of Na/K-ATPase enzymatic function, signaling axis, and NKA α-isoform expression in WT and A420P hiPSC and iCM. (**A**) Na/K-ATPase activity was measured in membrane preparations from hiPSC-WT and hiPSC-A420P (*n* = 4, ns: no significant difference found by unpaired Student’s *t*-test). (**B**) NKA α1-3 expression in cell lysates from iCM-WT and iCM-A420P normalized to GAPDH (*n* = 3, ns: no significant difference found by unpaired Student’s *t*-test). (**C**) Phosphorylated forms of ERK1/2 and Src normalized to total ERK1/2 and c-Src, respectively (*n* = 3). * *p* < 0.05, ** *p* < 0.01. Unpaired Student’s *t*-test.

As shown in Figure 3A, total Na/K-ATPase activity was comparable in cell membrane preparations obtained from hiPSC-WT and hiPSC-A420P. This finding is consistent with the previous observation that, when expressed in porcine epithelial cells (LLC-PK1), rat NKA α1 WT and A420P have comparable Na/K-ATPase enzymatic properties [58]. In the LLC-PK1 system, rat NKA α1 WT and A420P protein levels were also comparable. Accordingly, NKA signaling could be readily compared in LLC-PK1 expressing A420P vs. WT under preserved total cellular Na/K-ATPase function. In contrast to the epithelial LLC-PK1 system, in which only one NKA α isoform accounts for the total cellular Na/K-ATPase activity, three isoforms of the NKA α catalytic subunit (α1-3) support Na/K-ATPase activity in human cardiac myocytes [1,2,36]. As shown in Figure 3B, all isoforms were detected in both iCM lines. Importantly, isoform expression levels did not significantly differ between iCM-WT and iCM-A420P. In contrast, baseline levels of p-Src and p-ERK1/2 were significantly decreased in hiPSC-A420P compared to WT (Figure 3C), consistent with a substantial downregulation of the NKA α1/Src signaling axis in iCM-A420P. Therefore, it was concluded that the human WT/A420P iCM system, with intact NKA isoform expression and enzymatic activity, is a highly suitable system for studying the impact of the loss of the NKA α1/Src regulatory function on cardiac mitochondrial metabolic function and ROS production.

### 3.3. Mitochondrial Function in iCM-WT and iCM-A420P

**Figure 4:**
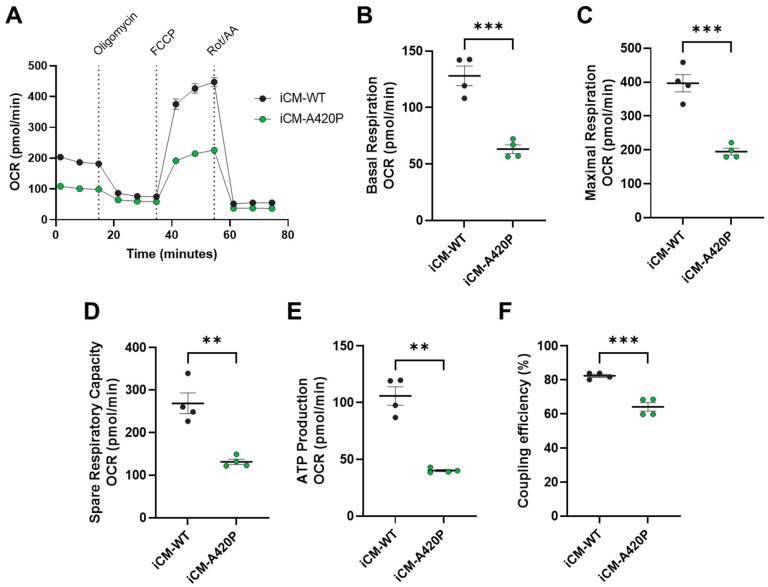
Seahorse metabolic analysis of iCM-WT and iCM-A420P. (**A**) Representative trace of a mitochondrial stress test in iCM-WT (black) vs. iCM-A420P (green). (**B**) Basal mitochondrial respiration. (**C**) Maximal mitochondrial respiration. (**D**) Spare respiratory capacity. (**E**) ATP production. (**F**) Coupling efficiency. OCR: oxygen consumption rate. FCCP: carbonyl cyanide-p-trifluoromethoxyphenylhydrazone; Rot/AA: Rotenone/Antimycin A. *n* = 4 independent experiments/group. ** *p* < 0.01, *** *p* < 0.001. Unpaired Student’s *t*-test.

Unlike the WT form, rat NKA α1 A420P mutant fails to maintain mitochondrial metabolic function when expressed in LLC-PK1. As we reported recently in Kutz et al. [56], this was reflected by decreased maximal mitochondrial respiration and spare respiratory capacity compared to the WT NKA α1-expressing cell line. As summarized in Figure 4, our studies using real-time cell metabolic analysis (Agilent Seahorse Extracellular Flux Analyzer) revealed that mitochondrial metabolic function was also reduced in iCM-A420P. Specifically, significant reductions of basal mitochondrial respiration (Figure 4B), maximal mitochondrial respiration (Figure 4C), spare respiratory capacity (Figure 4D), ATP production (Figure 4E), and coupling efficiency (Figure 4F) were detected.

### 3.4. Decreased ROS Production and Regulation of Gene Markers of Cardiac Oxidative Stress in iCM-A420P

**Figure 5:**
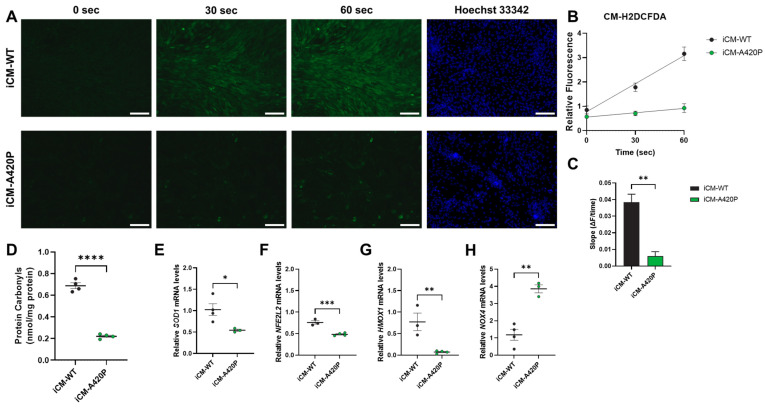
ROS production in iCM-WT and iCM-A420P. (**A**) ROS production was monitored over time in live iCM by oxidation of CM-H_2_DCFDA using a fluorescence microscope (Ex./Em.: 488/510 nm), with nuclear counterstaining by Hoechst 33342 (Ex./Em.: 359/457 nm). Scale bar: 200 µm. (**B**) Fluorescence relative to iCM-WT at 0 s (*n* = 3/genotype). (**C**) ROS production rate derived from the slope of the fluorescence curves (ΔF/time). (**D**) Protein carbonylation levels determined by ELISA (*n* = 4 independent samples/group). (**E**–**H**) RT-qPCR analyses of the genes encoding (**E**) superoxide dismutase 1 (*SOD1*), (**F**) NFE2-like bZIP transcription factor 2 (*NFE2L2*), (**G**) heme oxygenase 1 (*HMOX1*), and (**H**) NADPH oxidase 4 (*NOX4*) are shown. Relative mRNA expression was calculated according to the 2 (−ΔΔCt) method, using hypoxanthine-guanine phosphoribosyltransferase 1 (*HPRT1*) as the reference gene (*n* = 3–4 independent samples/group). * *p* < 0.05, ** *p* < 0.01, *** *p* < 0.001, **** *p* < 0.0001. Unpaired Student’s *t*-test.

Mammalian cells, including CM, have evolved strategies to regulate ROS levels to maintain physiological processes and survival. Low to moderate levels of ROS contribute to important functions, such as cell differentiation, growth, or apoptosis [62,63,64,65,66]. In contrast, a variety of pathological states, including cardiovascular disease, are associated with elevated ROS levels. NKA interaction with cardiotonic steroids (CTS) has been shown to activate several intracellular signaling pathways [67]. In the heart and other tissues, this activation can lead to ROS generation, which, in turn, positively feedbacks to the NKA in a ROS amplification loop [68,69]. Our recent RNA-seq analysis of hearts from mice genetically engineered to express a NKA α1 isoform with increased sensitivity to endogenous CTS suggests that this mechanism is physiologically relevant, and could have a profound impact on cardiac remodeling [55]. However, the design of the Marck et al. study in a global mutant mouse model did not allow us to determine whether the underlying mechanism occurred in the CM and whether it was Src-dependent. Accordingly, to assess whether NKA α1/Src regulatory function modulates ROS production in the CM, the redox status was compared in iCM-WT and iCM-A420P. ROS production assessed by fluorescence imaging of oxidized CM-H_2_DCFDA in live cells was drastically downregulated in iCM-A420P (Figure 5A–C). Protein carbonylation, a marker of oxidative damage, was also reduced in iCM-A420P (Figure 5D). Regulations at the transcriptional level were observed for SOD1, NFE2L2, and HMOX1, which belong to the cardiac antioxidant system and are important mediators of cardioprotection [42,70]. These genes were downregulated in iCM-A420P compared to iCM-WT (Figure 5E–G). In contrast, mRNA levels for NOX4, one of the main sources of ROS production in the heart [42,70], were upregulated (Figure 5H).

## 4. Discussion

Maintenance of a high ATP production and turnover rate by the cardiac myocyte is critical to ensure continuous cardiac contractile function. Adding to mounting evidence that an NKA α1/Src signaling axis regulates mitochondrial function and redox status in mammals [56], this study provides the first genetic evidence that NKA/Src tonically stimulates mitochondrial metabolic function and ROS production in human cardiac cells.

Gain/loss of function approaches in the porcine epithelial cell line LLC-PK1 have identified key functional sites on the NKA α1 polypeptide that form the structural basis for some of its isoform-specific receptor functions [59]. Using CRISPR/Cas9-mediated mutagenesis of selected functional sequences, we have subsequently generated functional hiPSC mutants of NKA α1. These included F97A/F100A (CBM), which disrupts NKA/caveolin 1 interaction, and A420P, which disrupts the “NaKtide” sequence critical for the NKA/Src regulatory axis. This line of investigation has revealed a fundamental role of the non-enzymatic functions of NKA in cell lineage specification, with a hierarchical organization of the associated functional domains. Hence, while the CBM mutation severely impaired myogenesis in vitro and halted somitogenesis in the mouse embryo [35], defects secondary to A420P mutation were less dramatic on skeletal muscle differentiation in vitro [57]. The present study suggests that the A420P mutation does not prevent the differentiation of hiPSC into cardiac myocytes, providing a clinically relevant model that also expresses multiple NKA α isoforms, and allowed us to investigate the specific role of the NKA/Src signaling axis in CM physiology.

Our previous studies have shown that the A420P mutant expressed in the pig LLC-PK1-derived cell line does not affect the expression of NKA or its ATP hydrolytic activity [58]. Similarly, the A420P mutation did not affect NKA enzymatic activity in hiPSC (Figure 3), and the expression of NKA α1-3 isoforms in iCM-A420P was not significantly different from the iCM-WT (Figure 3). However, we observed a drastic downregulation of both p-Src and p-ERK1/2 levels in the mutant hiPSC-A420P (Figure 3), which contrasts with the phenotype of LLC-PK1-expressing rat A420P α1. Indeed, in the LLC-PK1 model, A420P results in an increased of total cellular p-Src levels at baseline, consistent with the loss of regulatory control of Src kinase domain [58]. Elevated p-Src levels at baseline are also observed in LLC-PK cells expressing NKA α2 or α3 in the absence of α1 [59,71]; therefore, NKA α2 or α3 are not believed to fully support this α1-like Src kinase domain regulatory properties. Consistently, under genetic suppression of NKA α1, skeletal muscle α2 did not compensate α1-like regulation of the kinase in the mouse [56]. Although we consider it unlikely, further studies could reveal that cellular assembly of NKA A420P, α2, and/or α3 yields a NKA complex that enables baseline Src regulation, even in the LLC-PK1 model. Certainly, the striking difference between baseline p-Src levels in hiPSC and LLC-PK1 cells expressing the A420P α1 mutant underscores the importance of cell-specific attributes and the need for extensive studies of lineage-specific roles of NKA signaling.

**Figure 6:**
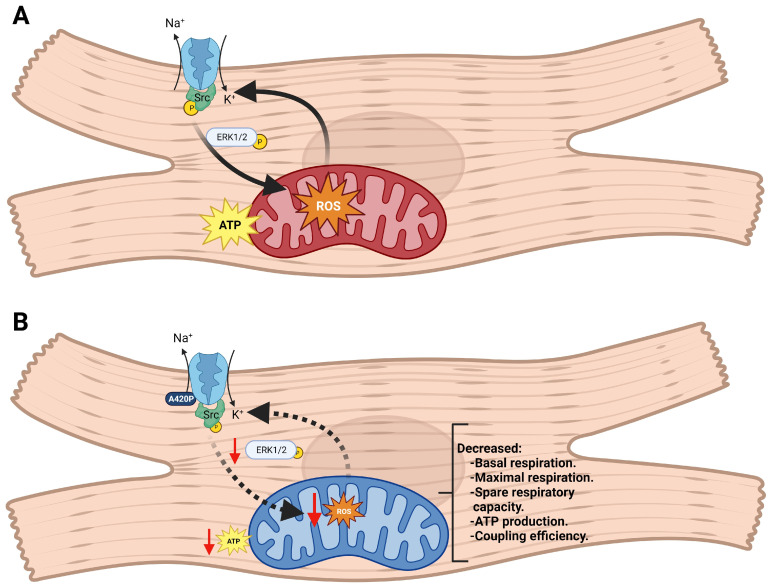
Na/K-ATPase α1/Src regulation of mitochondrial function and redox signaling in iCM. (**A**) In iCM-WT, the NKA ion-pumping function operates and the NKA/Src signaling tonically stimulates the phosphorylation of Src and ERK1/2, baseline mitochondrial metabolic function, and ATP production. Balanced ROS production originating from mitochondrial activity contributes to physiological baseline stimulation of NKA/Src through a feedforward mechanism. (**B**) In iCM-A420P, NKA activity is preserved. However, blunted NKA/Src signaling results in reduced phosphorylation of Src and ERK1/2, decreased basal respiration, maximal respiration, spare respiratory capacity, ATP production, coupling efficiency, and decreased ROS production. Created with BioRender.com (accessed on 31 October 2023).

According to the findings presented here, the NKA/Src signaling axis modulates the balance of oxidants, which is a potent regulator of cellular activity. Physiological levels of ROS modulate key cell processes such as excitation–contraction coupling, cell differentiation, cell proliferation, and metabolic pathways [72,73]. In contrast, unbalanced ROS production can lead to oxidative stress commonly found in cardiovascular diseases [62,63,64,65,66]. CTS-induced NKA signaling can lead to ROS production, which perpetuates NKA signaling activation through a positive amplification loop [68,69]. The generation of hydrogen peroxide by treating LLC-PK1 cells with glucose oxidase leads to Src and ERK1/2 phosphorylation, whereas the A420P mutant cannot respond to glucose oxidase treatment [74]. In good agreement, iCM-A420P have a drastic decrease in ROS production and protein carbonylation levels compared to iCM-WT (Figure 5), which is presumably related to low basal p-Src levels and a reduced NKA/Src/ROS amplification loop. Mechanistically, Liu et al. [67] have shown that cardiac myocytes pretreated with myxothiazol (a competitive inhibitor of ubiquinol) and diphenyleneiodonium (DPI—an inhibitor of flavoenzymes) can block ouabain-induced ROS production, suggesting that ROS derived from the electron transport chain in the mitochondria might be one of the sources of ROS produced by ouabain treatment. Along with our present study, these data suggest a direct link between CTS-induced ROS production through NKA signaling and mitochondrial activity. Our working model (Figure 6) proposes that the A420P mutation downregulates cardiac mitochondrial metabolic function, thereby reducing one of the main sites for CTS-induced ROS production.

The proposed mechanism of control of mitochondrial metabolic function by the NKA/Src signaling axis might be a new target for therapeutic intervention in cardiovascular diseases. Indeed, mitochondrial abnormalities have long been described in the setting of cardiomyopathies and heart failure, but recent studies propose that changes in cardiac metabolism are a cause, rather than an effect, of adverse cardiac remodeling [75,76,77], and that targeting this defect in a timely manner can be beneficial to treat cardiovascular diseases. We have previously demonstrated the importance of NKA α1/Src interaction for metabolic reserve in renal proximal tubule cells [56]. LLC-PK1-derived cells lines harboring the A420P mutation on NKA α1 demonstrated reduced maximal mitochondrial respiration and spare respiratory capacity, and a metabolic switch, being more reliant on glycolysis (higher glycolysis rate but no glycolytic reserve) than oxidative phosphorylation. Here, we show that the A420P mutation in iCM also induces a functional mitochondrial defect, reducing the maximal mitochondrial respiration and spare respiratory, ATP production, and coupling efficiency of these cells. In addition, heme oxygenase-1 (*HMOX1* gene) which is reduced in iCM-A420P, has been shown to affect mitochondrial biogenesis and quality control [78,79], and therefore might be associated with the metabolic phenotype of the iCM-A420P. Others have recently observed the importance of NKA/Src signaling in the regulation of cardiac mitochondrial metabolism and cardiac remodeling. Staehr et al. [47] focused on the cardiac phenotype of a mouse model of familial hemiplegic migraine type 2 (FHM2)-associated G301R mutation in the *Atp1a2* gene, which encodes the NKA α2 isoform. This mutation leads to migraine aura, a known risk factor for heart disease. In the heterozygous FHM2 mouse, increased cardiac expression of NKA α1 isoform was associated with left ventricular dilation and reduced ejection fraction at 8 months. Notably, exacerbated NKA-dependent Src kinase/Ras/ERK1/2 (p44/42 mitogen-activated protein kinase) signaling was observed, which is associated with mitochondrial uncoupling, increased oxidative stress, and a heart failure-associated metabolic shift. These effects led the authors to suggest that the *Atp1a2* mutation in FHM2 leads to disturbed cardiac metabolism and reduced cardiac function mediated via NKA α1-dependent ROS signaling through the Src/Ras/ERK1/2 pathway.

Hence, direct and indirect evidence from our group and others suggest that NKA/Src signaling tonically controls cardiac mitochondrial metabolic function and redox signaling. While chronic/dysregulated overstimulation disturbed cardiac metabolism in Staehr et. al. [47], physiological levels of activation maintained cardiac mitochondrial activity and ROS levels in the present study. The role of a favorable activation of this new branch of the NKA/Src-dependent axis in NKA-dependent cardioprotection [80,81] remains to be explored. Perhaps just as importantly, the potential role of abnormal/chronic overstimulation of this new NKA signaling branch in adverse cardiac remodeling warrants further investigation [82,83].

## Data Availability

The data presented in this study are available upon reasonable request from the corresponding author.

## Supplementary Materials

The following supporting information can be downloaded at: https://www.mdpi.com/article/10.3390/biomedicines11123207/s1, Table S1: Nanodrop analysis of RNA (Figure 1, Figure 2 and Figure 5). Data are represented as mean ± SEM; Table S2: Primer sequences used in RT-qPCR; Table S3: Volumes and concentrations of injections for Seahorse analysis, with final concentrations in parentheses; Video S1: Beating iCM-WT. Video S2: Beating iCM-A420P.
