# Supplementary material for: The Na/K-ATPase α1/Src Signaling Axis Regulates Mitochondrial Metabolic Function and Redox Signaling in Human iPSC-Derived Cardiomyocytes
Source: Biomedicines. 2023 Dec 2;11(12):3207. doi: 10.3390/biomedicines11123207 (PMC10740578; doi:10.3390/biomedicines11123207)
Supplement: Supplementary file 1 [file biomedicines-11-03207-s001.zip › Supplementary Materials-Biomedicines.pdf]

Supplementary Materials

Table S1. Nanodrop analysis of total RNA (Figures 1, 2, and 5). Data are represented as mean ± SEM.

	**Concentration (ng/µL)**	**260/280 ratio**
**hiPSC-WT (*N*=4)**	438.00±16.57	2.06±0.01
**hiPSC-A420P (*N*=4)**	418.75±29.78	2.06±0.01
**iCM-WT (*N*=4)**	374.50±69.48	2.04±0.03
**iCM-A420P (*N*=4)**	441.75±34.67	2.05±0.01

Table S2. Primer sequences used in RT-qPCR.

**Primer Name**	**Primer Sequence (5'→3')**
Human *HPRT1* Forward	TGGACAGGACTGAACGTCTT
Human *HPRT1* Reverse	TCCAGCAGGTCAGCAAAGAA
Human *MYH6* Forward	CTCCGTGAAGGGATAACCAGG
Human *MYH6* Reverse	TTCACAGTCACCGTCTTCCC
Human *TNNT2* Forward	CTGCTGTTCTGAGGGAGAGC
Human *TNNT2* Reverse	CACCAAGTTGGGCATGAACG
Human *SOD1* Forward	ACTGGTGGTCCATGAAAAAGC
Human *SOD1* Reverse	GGGCGATCCCAATTACACCA
Human *NFE2L2* Forward	AGGTTGCCCACATTCCCAAA
Human *NFE2L2* Reverse	ACGTAGCCGAAGAAACCTCA
Human *HMOX1* Forward	CTCTACCGCTCCCGCATGAAC
Human *HMOX1* Reverse	AAGCCCTGGTGCCCGTGAG
Human *NOX4* Forward	GGGAAGAGGTCATTTTCCGTGA
Human *NOX4* Reverse	GAGCCAGATGAACCCAAGCA

Table S3. Volumes and concentrations of injections for Seahorse analysis, with final concentrations in parentheses.

	**Mito Stress Test**
**Injection 1**	20 µl of 25 µM Oligomycin (2.5 µM)
**Injection 2**	22 µl of 20 µM FCCP (2.0 µM)
**Injection 3**	25 µl of 5 µM Rotenone/Antimycin A (5 µM)

Video S1: Beating iCM-WT.

Video S2: Beating iCM-A420P.
